# Sphingosine-1-Phosphate Receptor 2 Promotes Renal Microvascular Constriction and Kidney Injury Following Renal Ischemia-Reperfusion in Rats

**DOI:** 10.1093/function/zqaf024

**Published:** 2025-06-06

**Authors:** Zhengrong Guan, Colton E Remedies, Yanfeng Zhang, Paul W Sanders, Edward W Inscho, Wenguang Feng

**Affiliations:** Division of Nephrology, Department of Medicine, Heersink School of Medicine, University of Alabama at Birmingham, Birmingham, Alabama 35294, USA; Division of Nephrology, Department of Medicine, Heersink School of Medicine, University of Alabama at Birmingham, Birmingham, Alabama 35294, USA; Department of Genetics, Heersink School of Medicine, University of Alabama at Birmingham, Birmingham, Alabama 35294, USA; Division of Nephrology, Department of Medicine, Heersink School of Medicine, University of Alabama at Birmingham, Birmingham, Alabama 35294, USA; Department of Veterans Affairs Medical Center, Birmingham, Alabama 35294, USA; Division of Nephrology, Department of Medicine, Heersink School of Medicine, University of Alabama at Birmingham, Birmingham, Alabama 35294, USA; Division of Nephrology, Department of Medicine, Heersink School of Medicine, University of Alabama at Birmingham, Birmingham, Alabama 35294, USA

**Keywords:** sphingosine-1-phosphate, afferent arteriole, sphingolipid metabolites, JTE-013, SEW2871, sphingosine

## Abstract

Ischemia-reperfusion (IR) induced acute kidney injury (AKI) features increased renal vascular resistance, which is predominantly regulated by adjustments in afferent arteriolar diameter. Sphingosine-1-phosphate (S1P), a bioactive sphingolipid metabolite, is a potent vasoconstrictor in afferent arterioles. We hypothesized that IR enhanced afferent arteriolar sensitivity to S1P-induced vasoconstriction, thus contributing to renal microvascular dysfunction and kidney injury in AKI. The impact of IR on afferent arteriolar reactivity to S1P was assessed using the *in vitro* blood-perfused juxtamedullary nephron preparation in male rats subjected to 60 min of bilateral renal arterial ischemia followed by 24 h of reperfusion. Baseline diameter of afferent arterioles declined significantly following IR. S1P evoked concentration-dependent vasoconstriction in both sham and IR rats. However, the S1P concentration-response curve left-shifted after IR and its EC_50_ reduced by 8-fold (*P* < 0.05), suggesting enhanced afferent arteriolar reactivity to S1P. S1P receptor 2 (S1PR2) blockade with JTE-013 increased arteriolar diameter by 38 ± 7% following IR contrasted to a 9 ± 3% increase in sham rats (*P* < 0.05), indicating that endogenous S1P exerts a significant impact on afferent arteriolar tone after IR. Furthermore, IR upregulated mRNA and protein of S1PR2 in isolated preglomerular microvessels and elevated S1P content in kidney homogenates. Conversely, following IR, vasoresponsiveness to S1PR1 agonist, sphingosine, endothelin-1, norepinephrine, and angiotensin II did not differ from sham controls. JTE-013 treatment reduced plasma creatinine, tubular damage, and kidney ROS accumulation in IR rats. These data establish that IR enhances renal microvascular S1P-S1PR2 signaling and promotes kidney sphingolipid metabolites that could negatively affect kidney tissue perfusion, leading to AKI.

## Graphical Abstract

**Graphical Abstract:**
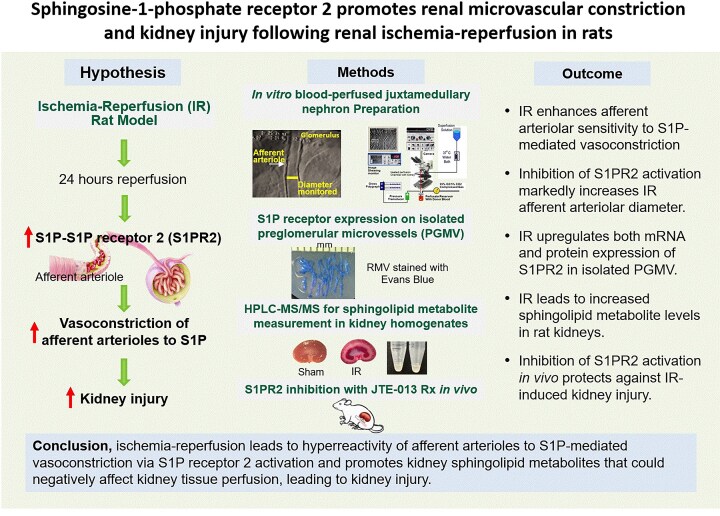

## Translational Statement

Afferent arterioles are the crucial preglomerular microvascular segments that regulate renal blood flow and glomerular hydrostatic pressure. This study reveals that renal ischemia-reperfusion (IR) enhanced S1P-mediated vasoconstriction of juxtamedullary afferent arterioles via S1P receptor 2 activation and increased kidney sphingolipid metabolites. The hyperreactivity of afferent arterioles to S1P-mediated vasoconstriction could represent a pathophysiological mechanism leading to the persistent increase of renal vascular resistance and kidney hypoxia in IR. Inhibiting S1P receptor 2 activation may serve as a therapeutic target for mitigating IR-induced kidney injury.

## Introduction

Renal ischemia-reperfusion injury (IR) is one of the leading causes of acute kidney injury (AKI), a significant unsolved clinical problem with high morbidity and mortality.^1^ The hallmarks of IR-induced renal hemodynamic alterations are tubular injury, increased renal vascular resistance (RVR) and reduced glomerular filtration rate (GFR) and renal blood flow (RBF).^2-6^ Pathophysiologic changes in kidney microcirculation play a critical role in the development of AKI and the AKI to chronic kidney disease (CKD) transition,^7-10^ but the pathological cellular or molecular signaling mechanisms underlying the increased RVR after IR are inconclusive. Afferent arterioles are the major resistance microvessels controlling renal vascular tone and regulating RBF and GFR. Afferent arteriolar reactivity is influenced by a variety of vasoconstrictors and vasodilators.^11-13^ Inappropriate adjustments of afferent arteriolar reactivity during IR could reduce RBF and oxygen delivery to kidney parenchyma and compound microvascular and epithelial injury.

S1P is a metabolite of sphingomyelin and is involved in diverse physiological and pathophysiological processes including cell proliferation and differentiation, angiogenesis and immune cell trafficking via activation of five S1P receptor (S1PR) subtypes, S1PR1-S1PR5.^14,15^ S1P has also emerged as an important mediator of vascular tone on both non-renal and renal resistance vessels.^16-22^ Our recent studies reveal that S1P is a potent vasoconstrictor of the preglomerular microvasculature (PGMV) in rats, predominantly afferent arterioles via activation of S1PR1 and S1PR2, while having no detectable effect on efferent arterioles.^21,23^ The exclusive and potent vasoconstrictor effect of S1P on afferent arterioles strongly implies a fundamental importance of S1P signaling in controlling glomerular capillary pressure and RBF. Accumulating evidence supports a critical role for S1P in the development of IR-AKI in mice but these studies were largely focused on tubular injury or endothelial permeability.^24-30^ The role for S1P in renal microvascular dysfunction, however, has never been addressed under IR conditions. We hypothesized that renal IR leads to enhanced sensitivity of afferent arterioles to S1P-mediated vasoconstriction, thus contributing to renal microvascular dysfunction and kidney injury in IR-induced AKI (IR-AKI).

In this study, we used the bilateral 60-min IR rat model which displays renal microvascular dysfunction in the early phase of IR^31,32^ and develops renal microvascular rarefaction^33^ and kidney fibrosis with persistent apoptosis^34^ in 3-4 wk. Moreover, this model is relevant to clinical setting in which IR develops, including major cardiac surgery, severe hemorrhage, intraoperative hypotension, and myocardial infarction.^35^ The in vitro blood-perfused juxtamedullary nephron (JMN) preparation permitted to direct assessment of afferent arteriolar responses to experimental manipulations by application of exogenous S1P, S1PR agonist or antagonist, S1P precursor, and other common vasoconstrictors associated with renal hemodynamic regulation. We determined mRNA and protein expression of S1PR on isolated PGMV. Moreover, we measured the major sphingolipid metabolites in kidney homogenates to determine the impact of IR on kidney sphingolipid metabolism. Finally, we also determined the impact of S1PR2 blockade on kidney function and injury.

## Methods

### Animal

A total of 265 male Sprague-Dawley rats weighing 300-400 g (Charles River Laboratories) were used. All animals were housed on a reversed 12-h light/12-h dark cycle and had *ad libitum* access to water and standard chow (PMI Nutrition International, LLC). All animals were maintained according to the National Institutes of Health Guide for the Care and Use of Laboratory Animals. All procedures were approved by the Institutional Animal Care and Use Committee at UAB.

### Rat IR Model

Kidney IR was induced by occluding both renal arteries for 60 min followed by 24 h of reperfusion as described previously.^31,32,36^ Under anesthesia with ketamine [100 mg/kg. body weight (BW)] and xylazine (10 mg/kg. BW) injection intraperitoneally (IP), rats were kept on a homeothermic controlled table to maintain body temperature at 36.5-37°C. Buprenorphine SR (1.2 mg/kg. BW) was given subcutaneously prior to surgery. After 60 min of ischemia, the clamps were removed and the reperfusion was confirmed by visual inspection of the kidney surface. After 24 h of reperfusion, the animals were re-anesthetized by ethyl-1-methylpropyl-thiobarbiturate (Inactin^®^, 100 mg/kg. BW, IP), and the right kidney was perfused for the JMN preparation. Some rats were prepared for kidney collection and PGMV isolation. Sham-operated rats served as controls. Rats were randomly assigned to each group and monitored closely post-surgery to ensure no severe distress (>20% weight lost). Group size was determined based on our previous studies with a power of 80% to detect an effective size of 1.5 statistic deviation or larger under the significance level of 0.05.^21,22,32,37-39^

### The *In Vitro* Blood-Perfused JMN in Rats

The JMN preparation was used for assessing afferent arteriolar reactivity as described previously.^21,22,32,37-39^ Two identical rats (blood and kidney donors) were used for one JMN preparation and only one afferent arteriole was assessed in each preparation. Blood was centrifuged and processed to remove white blood cell fraction and platelets. Plasma and washed erythrocytes were reconstituted to achieve a final hematocrit of ∼33%. The right renal artery was cannulated and perfused with Tyrode's buffer containing 5.2% bovine serum albumin (BSA). After completion of the microdissection, the kidney perfusate was switched from 5.2% BSA perfusate to the reconstituted blood. Afferent arteriole was identified by tracking RBF direction to the attached glomerulus. The tested drugs were delivered via superfusate (1% BSA-Tyrode’s buffer) onto the inner surface of the kidney via a multi-channel valve. At the conclusion of each experiment, the kidney was superfused with 55 mm KCl^32,38^ to exclude a general vascular failure of the contractile apparatus. The image of the vessels was displayed on a video monitor via a high-resolution NC-70 Newvicon video camera (DAGE-MTI) and recorded on digital video disk for later analysis. The inner arteriolar diameters were measured at 12-s intervals at a single site of the middle segment of afferent arterioles using an image-shearing monitor (Model 908, Vista Electronics) and averaged from all diameter measurements during the last 2 min of each 5-min treatment period.

### Experimental Protocols

After an initial equilibration period (>20 min) with the reconstituted blood perfusion at perfusion pressure of 100 mmHg, each experiment started with a 5-min control period to establish the steady state arteriolar diameter (Baseline).

### Experiment 1: Impact of IR on Afferent Arteriolar Responses to Exogenous S1P

After a 5-min baseline recording, the influence of S1P on afferent arteriolar reactivity was assessed in sham and IR kidneys by exposure to increasing S1P (ENZO Life Sciences, Inc.) concentrations over a log concentration scale (10^−10^ to 10^−5^ M, *n* = 7 kidneys/group). Each concentration of S1P was superfused for 5 min and the arteriolar diameter was measured.

### Experiment 2: Impact of IR on Afferent Arteriolar Responses to S1P Precursor, Sphingosine

Because biologically inert S1P analogs are not commercially available, we used sphingosine as a “physiologically inert negative control” to determine if the vasoconstriction observed is S1P specific or a non-specific effect of S1P. Similar to the S1P concentration-responses, after a 5-min baseline was recorded, sphingosine (Cayman Chemical, 10^−10^ to 10^−5^ M) was assessed in sham and IR kidneys (*n* = 6 kidneys/group).

### Experiment 3: Impact of IR on Afferent Arteriolar Responses to S1PR1 Activation

S1RP1 was detected in PGMV.^21^ To determine if the enhanced sensitivity of afferent arterioles to S1P is via S1PR1 activation, the concentration-response to the selective S1PR1 agonist, SEW2871 (10^−10^ to 10^−5^ M, Cayman Chemical) was assessed in sham and IR kidneys (*n* = 6 kidneys/group).

### Experiment 4: Effect of S1PR2 Antagonist on Afferent Arteriolar Diameter of IR Rats

We applied a selective S1PR2 blocker (JTE-013) to the inner cortical surface of kidneys to determine if the enhanced RVR in IR rats arose from S1PR2 activation, because specific pharmacological S1PR2 agonists were not available. After a 5-min baseline period, kidneys were exposed to JTE-013 (10^−5^ M, Tocris Bioscience) over 30 min. Three groups were studied: sham + JTE-013, IR + JTE-013 compared to the time-course in IR kidneys without JTE-013 (*n* = 6 kidneys/group).

### Experiment 5: Impact of IR on Afferent Arteriolar Responses to Endothelin-1 (ET-1), Norepinephrine (NE), and Angiotensin II (Ang II)

To verify if the enhanced vasoconstriction of afferent arterioles is unique to S1P or uniform to vasoconstrictors in IR rats, we assessed the vasoconstrictor properties of other G-protein coupled receptors using ET-1, NE, or Ang II. Briefly, after a 5-min baseline period, kidneys were exposed to increasing concentrations of ET-1 (10^−13^-10^−8^ M), NE (10^−8^, 10^−7^ and 10^−6 ^M), or Ang II (10^−12^-10^−7 ^M). Each JMN preparation was only used for one drug tested (*n* = 6 kidneys/group).

### PGMV Isolation for *S1pr* mRNA Expression

PGMV were isolated as described previously.^21,40,41^ Under anesthesia, abdominal aorta was cannulated for retrograde perfusion with physiological buffer solution (PBS) to flush out blood from kidneys. Medulla and intrarenal arteries were removed. Cortical tissue was gently pressed through a 100 μm nylon sieve (BioDesign, Inc.), and the retentate washed with ice-cold PBS. The vascular tissue on the sieve was transferred into RNAlater^™^ stabilization solution (Invitrogen, Thermo Fisher Scientific) and stored at −20°C to prevent mRNA degradation. Segments of arcuate and interlobular arteries with attached afferent arterioles, or PGMV, were identified and collected by microdissection using a stereoscope for mRNA extraction.

Total RNA was extracted from isolated PGMV or kidney cortical tissue homogenates with TRIzol (Invitrogen) and treated with DNAase I to remove genomic DNA and then purified with an RNA purification kit (Invitrogen). The DNA-free RNA was reverse transcribed to cDNA with use of the SuperScript IV RT Kit (Invitrogen). cDNA was amplified with SYBR Green PCR in the LightCycler^®^ 480 system (Roche Diagnostics) and specific primers (Supplementary Table 1) for 40 cycles. Steady-state mRNA levels were calculated according to threshold cycle generated with the LightCycler^®^ 480 software. Expression of each mRNA was normalized to 18 s and standardized to the sham group as 1.

### Western Blot Analysis for S1PR in Isolated PGMV

The isolation of PGMV was described previously.^21,40^ Similar to the aortic retrograde perfusion as mentioned above, but the kidneys were flushed with 5.2% BSA perfusate followed by 1% Evans blue. The renal cortical tissue was pressed through a 100 μm nylon sieve and rinsed with ice-cold PBS. The vascular tissue remaining on the sieve was transferred to a 20 mL-PBS containing albumin, dithiothreitol, collagenase type II and trypsin inhibitor (4 mg/each) for a 20-min incubation at 36.5°C. The vascular tissue was removed from the enzyme solution and transferred to a 70 μm nylon sieve where it was vigorously rinsed with ice-cold PBS. The sieve with the retained vascular tissue was transferred to a petri dish containing ice-cold PBS. Segments of interlobular artery with attached afferent arterioles were collected using a stereoscope and were stored at −80°C until analysis. Proteins (20 μg) were separated on a 4-12% Bis-Tris electrophoresis gel (Invitrogen) and were electrophoretically transferred onto nitrocellulose membranes. The membranes were blocked with 5% fat-free milk in Tris-buffered saline and were incubated with subtype-specific primary antibodies against S1PR1 (1:500, ab77076, Abcam), S1PR2 (1:500, sc-25491, Santa Cruz), and S1PR3 (1:500, ab108370, Abcam) overnight (4°C),^21^ respectively. The washed membranes were then incubated with donkey anti-rabbit IgG horseradish peroxidase conjugate (GE HealthCare). Densitometry was performed using enhanced chemiluminescence detection (Konica Corporation, Japan) and was normalized by *β*-actin expression using UN-SCAN-IT software (Silk Scientific, Inc.).

### Sphingolipid Metabolite Measurement

Endogenous sphingolipid metabolites were measured in kidney cortical and medullary tissue homogenates and plasma using the high-performance liquid chromatography-tandem mass spectrometry (HPLC-MS/MS) technologies at the Lipidomics Core Facility, Medical University of South Carolina (MUSC). Briefly, after 24 h of post-sham or IR (*n* = 4 rats/group), kidneys were perfused with PBS to flush out blood. Cortical and medullary tissue were separated and were immediately frozen in liquid nitrogen. The kidney tissues were homogenized with a tissue homogenation buffer containing 0.25 M sucrose, 25 mm KCl, 50 mm Tris, and 0.5 mm EDTA as described by Bielawski et al.^42^ Protein was measured by Bio-Rad protein assay (Bio-Rad Laboratories, CA, USA). Each kidney sample contained 1 mg protein in ∼ 100 µL and was shipped with dry-ice for analysis by the MUSC Lipidomics Core Facility team.

### Treatment With S1PR2 Blocker on Kidney Function, Histology, and Reactive Oxygen Species Accumulation in IR Rats

Because mRNA and protein expression of S1PR2 were increased in PGMV after IR, a set of rats was randomly treated without or with JTE-013 (*n* = 6-8 rats/each group). JTE-013 (0.1 mg/kg. BW, IP) was given 30 min prior to ischemia and were repeated at the time of reperfusion (IR + JTE-013). After 24 h of reperfusion, blood was collected, and the left kidneys were harvested and snap frozen in OCT blocks. The right renal artery was cannulated and flushed with PBS followed by a 4% paraformaldehyde solution. The fixed kidney sections (3-4 μm) were stained with hematoxylin and eosin (HE) for histopathological analysis.^31,39,43^ Plasma creatinine was assessed by picric acid assay based on the Jaffe reaction. Kidney injury was assessed in a blinded fashion in 10 randomly selected non-overlapping fields from the cortex using Paller's semiquantitative scale.^44^ Ten proximal tubule sections from each field were randomly scored. A score was given for loss of brush-border of the proximal tubule (1 point), cytoplasmic vacuolization (1 point), tubular epithelial cell flattening (1 point), interstitial edema (1 point), cell necrosis (1 or 2 points), cell membrane bleb formation (1 or 2 points), and tubular lumen obstruction (1 or 2 points). A count of zero indicated no evidence for these changes. The maximum score per field from the average of ten tubule sections was 10 and the total was 100 for each kidney, with higher scores representing more severe damage.

For the in situ detection of ROS, a new set of sham, IR and JTE-013 treated IR rats (*n* = 5-7 rats/each group) was prepared 24 h post-surgery for the snap-frozen kidney collection using a cryosectioning technique and fluorescence microscopy.^45-47^ Briefly, after the kidney was harvested and cut longitudinally into three portions, the middle portion was embedded in OCT followed by rapidly freezing on a dry-ice chilled isopentane bath. Cryosections (5-7 μm) were prepared immediately and were incubated with the fluorogenic probe, H_2_DCFDA (20 μm, Invitrogen) for 30 min at 37°C. Images were captured under the same exposure time (1 s) and ×20 magnification using a digital camera (Olympus DP12) attached to an Olympus BX40 microscope (Olympus America). The density of fluorescence staining was analyzed using ImageJ^48^ in a blinded fashion. Ten fields were randomly taken from renal cortex or outer medulla and averaged for each kidney.

### Statistical Analysis

All values are expressed as a mean ± SEM. Arteriolar diameter was presented as actual diameter and/or is normalized as percentage of baseline diameter. The concentration for a half-maximal vasoconstriction (EC_50_) of S1P was calculated by an objective approach using a sigmoidal curve (GraphPad PRISM Software). The comparisons within group were made by one-way ANOVA for repeated measures followed by post-hoc analysis with Dunnett's multiple range test. Statistical differences across multiple groups were determined using one-way ANOVA and Tukey's post-hoc test while unpaired *t*-test was only used for the comparisons between two groups. A *P* value < 0.05 was considered statistically significant.

## Results

### IR Enhanced Afferent Arteriolar Vasoconstriction to S1P

**Figure 1.**
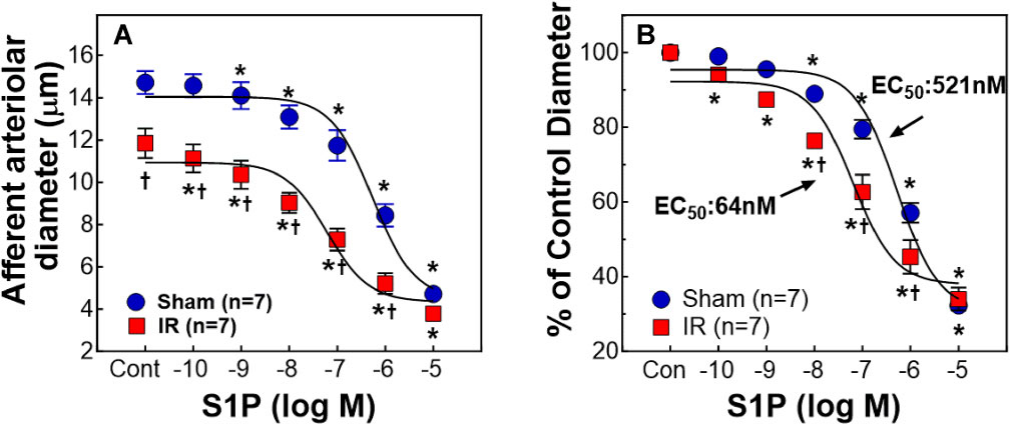
Impact of ischemia-reperfusion (IR) on afferent arteriolar reactivity to exogenous S1P. A: Afferent arteriolar response to S1P was assessed in male sham-operated (sham) and ischemia-reperfusion (IR) rats (*n* = 7/each group) using the *in vitro* blood-perfused juxtamedullary nephron preparation while perfusion pressure was held at 100 mmHg. B: The same data are normalized as a percentage of the baseline diameter [Control (Con)]. The EC_50_ was calculated and analyzed based on a non-linear regression with Sigmoidal dose-response (GraphPad Prism 10). IR left-shifted the S1P response-curve with a significant decline in EC_50_ (*P* < 0.05). Values are expressed as the mean ± SEM. For within-group analysis, a one-way repeated-measures ANOVA with a Dunnett's post hoc test was performed. *P < 0.05 vs. control diameter in the same group. Comparisons between groups were performed with unpaired *t*-test. †*P* < 0.05 vs. sham at the same concentration. *n* represents the numbers of rats.

Figure 1 illustrates the effect of exogenous S1P on afferent arteriole diameter of sham and IR rats after 24 h of reperfusion. Baseline arteriolar diameter significantly decreased in IR compared to sham rats (Figure 1A, 11.9 ± 0.7 vs. 14.7 ± 0.5 µm, *P* < 0.05). Superfusion of S1P evoked profound concentration-dependent vasoconstriction in both groups. The S1P response, however, was significantly enhanced in IR as evidenced by a leftward shift in the S1P concentration-response curve (Figure 1B). The EC_50_ of S1P (calculated using a sigmoidal dose-response curve) was significantly lower in IR than the EC_50_ in shams (64 vs. 521 nm, *P* < 0.05), indicating that IR afferent arterioles are more sensitive to S1P.

### Afferent Arteriolar Responses to Sphingosine or S1PR1 Agonist Were Unaltered by IR

**Figure 2.**
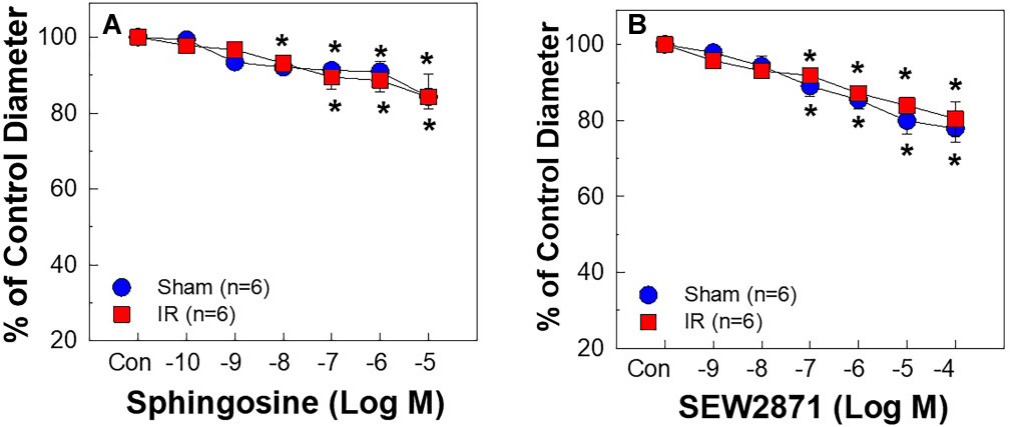
Impact of ischemia-reperfusion (IR) on afferent arteriolar reactivity to sphingosine and S1P receptor 1 (S1PR1) agonist. Afferent arteriolar responses to sphingosine (A, the precursor of S1P) or SEW2871 (B, a selective S1PR1 agonist) were assessed in male sham and IR rats. The data are normalized as a percentage of the baseline diameter [Control (Con)] for each group. No differences were detected between sham and IR. Values are expressed as the mean ± SEM. For within-group analysis, one-way repeated-measures ANOVA with a Dunnett's post hoc test was performed. *P < 0.05 vs. baseline diameter in the same group. *n* represents the numbers of rats.

Because of the lack of “biologically inert S1P analogs,” we used sphingosine as the S1P negative control. Superfusion of sphingosine to sham rats caused mild vasoconstriction of afferent arterioles, reducing the diameter to 85 ± 6% of the baseline at 10^−5^ M (Figure 2A). The sphingosine concentration profile was indistinguishable between sham and IR, suggesting that S1P acts through its specific S1PR activation.

We also determined afferent arteriolar response to S1PR1 agonist, SEW2871. Similar to our previous report,^21^ application of SEW2871 caused vasoconstriction in sham rats, reducing diameter to 78 ± 4% of the baseline at 10^−5^ M (Figure 2B). IR did not alter the SEW2871 concentration-response profile.

### S1PR2 Inhibition Markedly Increased Afferent Arteriolar Diameter in IR Rats

**Figure 3.**
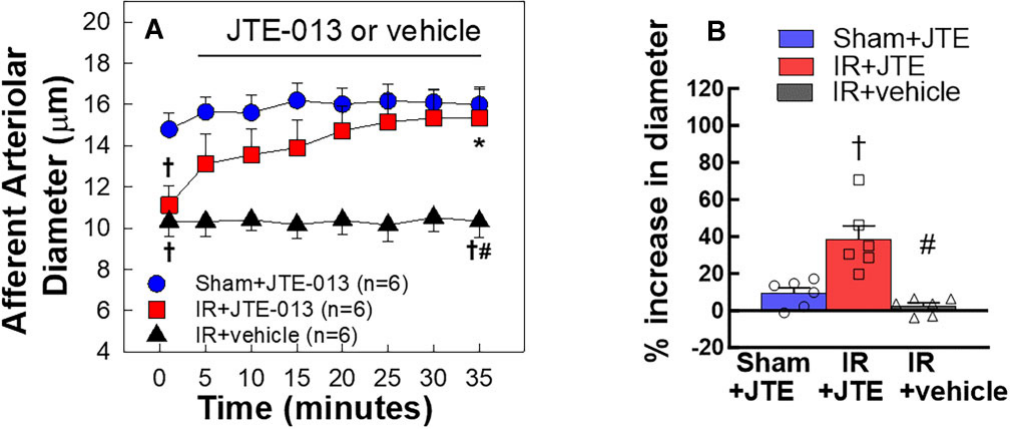
Impact of S1P receptor 2 (S1PR2) inhibition on afferent arteriolar diameter of ischemia-reperfusion (IR) rats. A: Inhibition of S1PR2 activation with JTE-013 (10 µm) on afferent arteriolar diameter was assessed in male sham and IR rats after 24 h of reperfusion. JTE-013 led to greater vasorelaxation in IR than the response in sham while IR afferent arteriolar diameter remained unchanged during superfusion of a vehicle. B: Data are expressed as the percentage increases in diameter at the end of JTE-013 superfusion. Values are means ± SEM (*n* = 6/group). For within-group analysis, one-way repeated-measures ANOVA with a Dunnett's post hoc test was performed. *P < 0.05 vs. baseline diameter in the same group. Comparisons between groups were performed with a one-way ANOVA and Tukey's post-hoc test with †*P* < 0.05 vs. sham rats and #*P* < 0.05 vs. IR rats. *n* represents the numbers of rats.

Figure 3A shows the impact of the selective S1PR2 antagonist, JTE-013, on baseline arteriolar diameter over a time-course. As expected, baseline diameter declined significantly in IR versus shams (*P* < 0.05). Acute exposure to JTE-013 (10^−5^ M) slightly increased the sham arteriolar diameter but dilated IR arterioles dramatically (Figure 3A). The diameter of afferent arterioles was almost completely recovered in the IR + JTE-013 group compared to the sham group after a 30 min superfusion with JTE-013 (15.3 ± 1.4 vs. 16.1 ± 0.7 µm). Figure 3B illustrates a 38 ± 7% of increase in IR contrasted to a 9 ± 3% increase in shams (Figure 3B, *P* < 0.05). Without JTE-013, the IR arteriolar diameter remained stable over the entire study. This suggests that endogenous S1PR2 activation exerts a greater influence on afferent arteriolar tone in IR than in shams.

### Afferent Arterioles From IR Rats Maintained Normal Vasoconstriction to ET-1, NE, or Ang II

**Figure 4.**
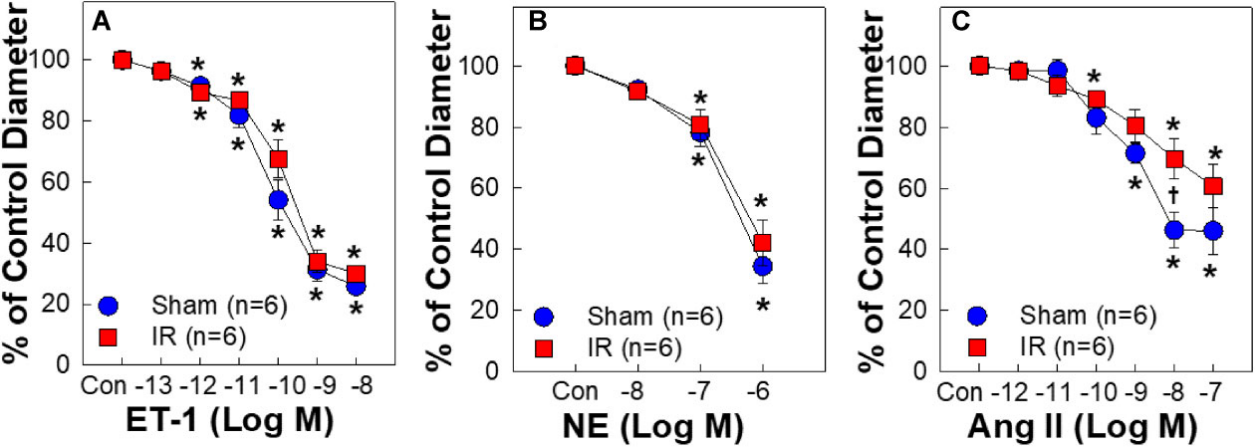
Afferent arteriolar responses to endothelin-1 (ET-1, A), norepinephrine (NE, B) and angiotensin II (Ang II, C) in male sham-operated (sham) or ischemia-reperfusion (IR) rats. Data are normalized by a percentage of the baseline diameter [control (Con)]. No difference was detected on ET-1, NE, or Ang II between sham and IR except an attenuated vasoconstriction to 10^−8^ M Ang II in IR. Values are means ± SEM (*n* = 6/per group in each set). For within-group analysis, one-way repeated-measures ANOVA with a Dunnett’s post hoc test was performed. *P < 0.05 vs. baseline diameter in the same group. Comparisons between groups were performed with unpaired *t*-test. †*P* < 0.05 vs. sham at the same concentration. *n* represents the numbers of rats.

To determine if the enhanced vasoconstriction is uniform to vasoconstrictors or is unique to S1P in IR kidneys, we assessed afferent arteriolar responses to the most common renal vasoconstrictors, ET-1, NE, and Ang II. Afferent arteriolar responses to ET-1 (Figure 4A) or NE (Figure 4B) were almost identical between sham and IR rats. The vasoconstrictor response to Ang II (Figure 4C) was also similar between the two groups except Ang II at 10^−8^ M where the vasoconstriction to Ang II was attenuated in IR (*P* < 0.05 vs. shams). These observations support that the enhanced vasoconstriction in our IR rat model is unique to S1P.

### IR Upregulated *S1pr2* Mrna and Its Protein Expression in Isolated PGMV

**Figure 5.**
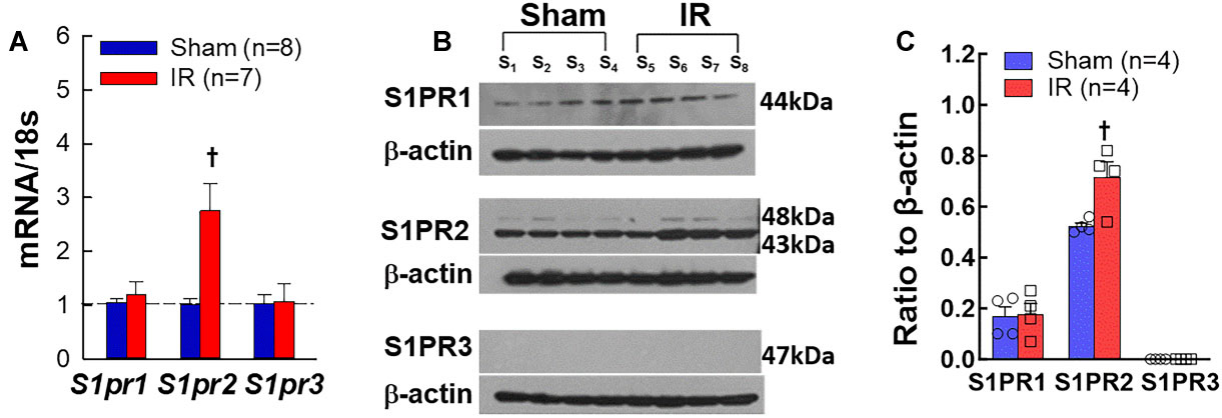
mRNA and protein expression of S1P receptors (S1PR) 1-3 in isolated preglomerular microvessels (PGMV) of sham-operated rats (sham) and rats subjected ischemia followed by 24 h of reperfusion (IR). A: The total mRNA of *S1pr2* was significantly increased in PGMV isolated from IR rats whereas the mRNA levels of *S1pr1* and *S1pr3* remained unchanged. B: Representative Western blot images for S1PR1, S1PR2, and S1PR3 expression in PGMV isolated from sham and IR rats. *β*-actin serves as a loading control and is shown in the bottom of each panel. C: densitometry analysis of S1PR protein expression. S1PR2 protein expression was significantly increased in IR PGMV. Values are means ± SEM. Comparisons between groups were performed with unpaired *t*-test. †*P* < 0.05 vs. sham for each S1PR. *n* represents the numbers of rats.

Figure 5 illustrates the mRNA and protein expression of three S1PR1, S1PR2, and S1PR3 in isolated PGMV of sham and IR rats 24 h of reperfusion. *S1pr2* mRNA expression increased significantly in PGMV of IR rats (Figure 5A). In contrast, both *S1pr1* and *S1pr3* mRNA expressions were unaltered in PGMV after IR. Figure 5B shows immunoblot images of S1PR protein expression in isolated PGMV from sham and IR rats. Consistent with mRNA expression, S1P2R protein expression was also significantly increased in IR PGMV (Figure 5C, *P* < 0.05) whereas S1PR1 was similar between IR and sham rats. Similar to our previous study,^21^ we did not detect S1PR3 from both groups. These results demonstrate that IR increased S1PR2 expression in PGMV.

### IR Increased Sphingolipid Metabolite Contents in Kidney Tissues

**Figure 6.**
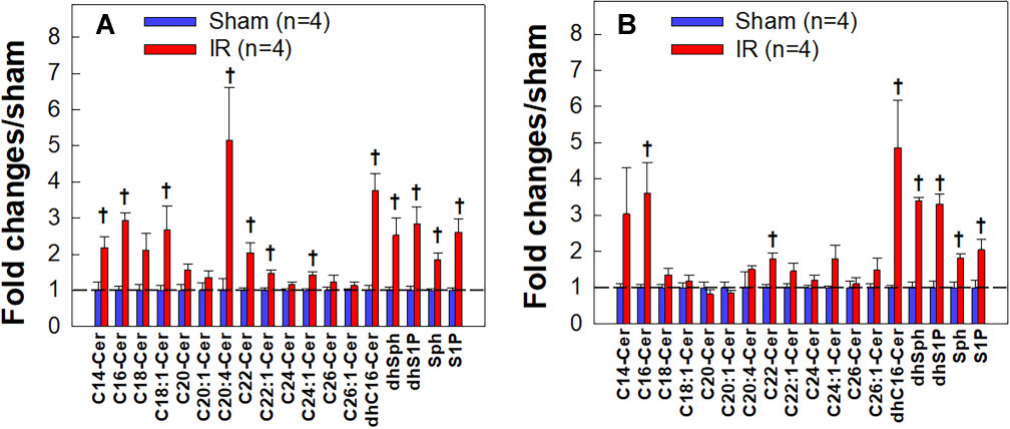
Sphingolipid metabolites are upregulated in kidney tissue homogenates of ischemia-reperfusion (IR) rats. The content levels of 18 endogenous sphingolipid metabolites in renal cortical (A) and medullar (B) tissue homogenates of sham and IR rats after 24 h of reperfusion, respectively, by using the liquid chromatography-mass spectrometry (LC/MS) method. The data are normalized by the respective sham contents. The data above 1 indicate increases. The raw data are provided in Supplementary Table 1. Values are expressed as means ± SEM. Comparisons between groups were performed with unpaired *t*-test. †*P* < 0.05 vs. sham for each metabolite. *n* = 4/each group. Cer: ceramide; dhSph: dihydrosphingosine; dhS1P: dihydrosphingosine-1-phosphate; Sph: sphingosine; S1P: sphingosine-1-phosphate.

Figure 6 represents a total of 18 sphingolipid metabolites measured in renal cortical and the outer medullary tissue homogenates at 24 h post-IR. The data are normalized by the respective sham contents, therefore, the data above 1 indicate increases in the sphingolipid metabolites. The raw data are provided in Supplementary Table 2. Among them, there were 12 and 8 sphingolipid metabolites that increased significantly in cortical (Figure 6A) or medullary (Figure 6B) homogenates of IR kidneys, respectively (*P* < 0.05). Importantly, S1P content was increased 2.6-fold from 1.2 ± 0.1 to 3.2 ± 0.5 pmol/mg protein and 2-fold from 1.8 ± 0.4 to 3.7 ± 0.5 pmol/mg protein in IR cortical or medullary homogenates, respectively (*P* < 0.05 vs. sham). In contrast, the plasma S1P concentration was not statistically different between IR and shams (77±8 vs. 91 ± 1 pmole/100 μL, *P* > 0.05, Supplementary Table 2). These results suggest that IR stimulates kidney sphingolipid metabolism.

### Treatment With the S1PR2 Blocker Reduced Plasma Creatinine, Tubular Injury, and Kidney ROS Accumulation in IR Rats

**Figure 7.**
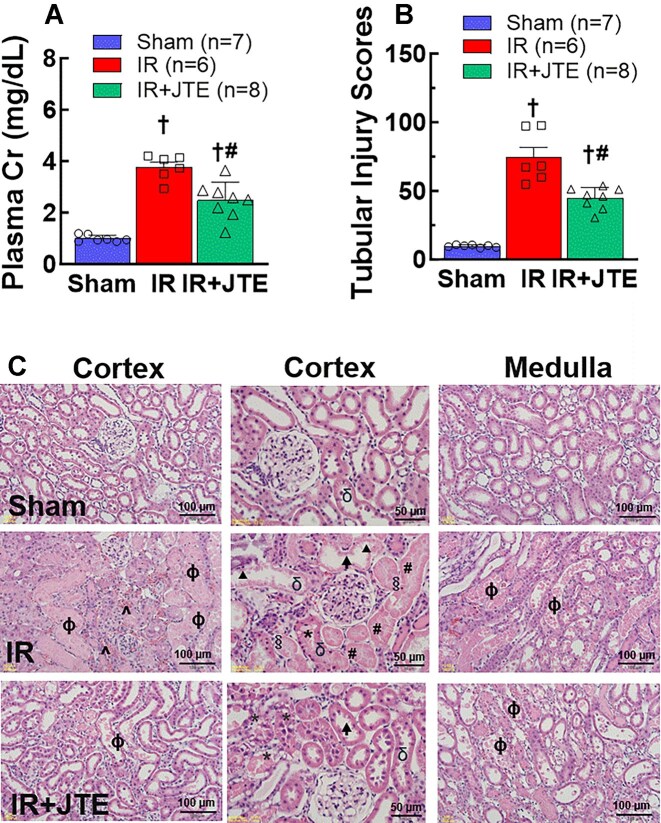
Inhibition of S1P2 receptor activation on kidney function and proximal tubular injury in renal ischemia-reperfusion (IR) rats after 24 h of reperfusion. A: Treatment with a S1PR2 blocker, JTE-013 (JTE), reduced plasma creatinine (Cr) concentration in IR rats. B: JTE-013 treatment reduced proximal tubular injury as scored using Paller's semiquantitative scale^44^ as described in the text. The total injury score was counted in 10 fields per kidney. C: Representative findings of proximal tubular changes of renal cortex and medulla from sham (Top panels), IR (Middle panels), and JTE-013 treated IR (IR + JTE, bottom panels) rats, respectively. Formation of casts in tubular limens (ɸ phi), cytoplasmic vacuolization (* asterisk), tubular necrosis (# hash signs), cell swelling (§ silcrow), sloughed cells in tubular lumens (δ delta), loss of brush-border (↑ arrow), flattening of tubular epithelium (▴ arrowhead), and trapping of erythrocytes (^ circumflex). Values are means ± SEM. Statistical analysis was performed using one-way ANOVA followed by a Tukey's post-hoc test. †*P* < 0.05 vs. sham; #*P* < 0.05 vs. IR alone rats. *n* represents the numbers of rats.

**Figure 8.**
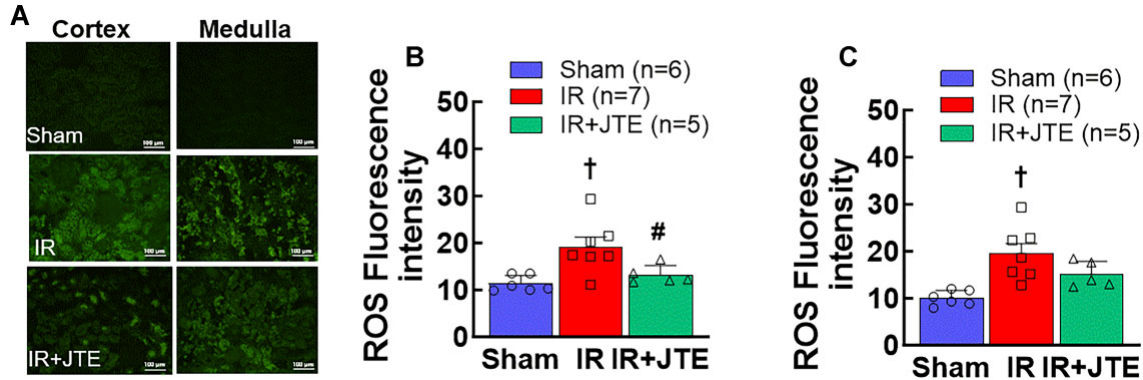
Inhibition of S1P receptor 2 activation reduces ROS accumulation in ischemia-reperfusion (IR) rat kidneys after 24 h of reperfusion. A: Representative fluorescence images using the fluorogenic probe, H_2_DCFDA, in renal cortical and medullary regions of sham, IR and IR rats treated with JTE-013 (IR + JTE), a selective S1PR2 inhibitor. B and C: Quantification of fluorescence intensity in renal cortical and medullary regions, respectively. A total of 10 fields per kidney were randomly selected. Values are expressed as means ± SEM. Statistical analysis was performed using one-way ANOVA followed by a Tukey's post-hoc test. †*P* < 0.05 vs. sham; #*P* < 0.05 vs. IR alone rats. *n* represents the numbers of rats. White bar indicates 100 µm.

As expected, plasma creatinine concentration was markedly elevated in IR rats (3.7 ± 0.2 vs.1.2 ± 0.1 mg/dL in sham, Figure 7A, *P* < 0.05) but significantly lower in IR treated with JTE-013 (2.4 ± 0.3 mg/dL, *P* < 0.05), albeit it was still high compared to sham rats (*P* < 0.05). Morphological analysis (Figure 7B&C) revealed that severe cell damage was detected in IR kidneys, including cell swelling and necrosis, loss of brush-border of the proximal tubule, cellular vacuolization, and the presence of luminal casts and sloughed cells in proximal tubules. The tubular injury score was significantly higher in IR kidneys than the score in sham kidneys (67.6 ± 7.6 vs. 9.9 ± 0.3, Figure 8B, *P* < 0.05). The extent of tubular injury was significantly decreased in JTE-013 treated IR kidneys. The tubular structure was better preserved as evidenced by less necrosis and cell damage in the proximal tubules and the reduced injury score (46.8 ± 2.6, *P* < 0.05), although there were still some cell casts and necrosis in the kidneys. Figure 7C represents light microscopic images of renal cortex and medulla from sham, IR and JTE-013 treated IR rats, respectively. There was significant trapping of erythrocytes in the IR medulla but less in JTE-013 treated IR kidney. These data suggest that S1PR2 blockade mitigates kidney injury, consistent with S1P being involved in IR-induced AKI.

Increased ROS accumulation plays an important role in the development of renal microvascular dysfunction after IR.^32,49^ Figure 8A represents the fluorescence images in kidney sections using the fluorogenic probe, H_2_DCFDA. The fluorescence intensity markedly increased in IR cortex (18.7 ± 2.4 vs. 10.9 ± 0.7 in sham, Figure 8B, *P* < 0.05) and in medulla (19.0 ± 2.5 vs. 9.6 ± 0.7 in sham, Figure 8C, *P* < 0.05), respectively. JTE-013 treatment reduced both cortical and medullary ROS levels to values that were not different from sham (13.5 ± 1.1 and 15.4 ± 1.5, *P* > 0.05 vs. sham, respectively). These data suggest that S1PR2 activation increased ROS accumulation in IR kidneys.

## Discussion

Early studies established a critical role for S1P in IR-associated tubular injury in mice.^24-30^ S1P is a strong vasoconstrictor of preglomerular microvasculature.^16,21^ The current study provides compelling evidence that S1P might also play an important role in the pathological microvascular alterations in IR rats. We reveal that afferent arterioles of IR rats exhibited hyperreactivity to exogenous S1P-mediated vasoconstriction as evidenced by a leftward shift of S1P concentration-response curve with an 8-fold reduction in EC_50_. Afferent arteriolar responses to the S1P precursor, sphingosine, and the specific S1PR1 agonist, SEW2871, were minimal and remained unaltered by IR. Acute exposure to the S1PR2 inhibitor, JTE-013, markedly increased afferent arteriolar diameter in IR rats, suggesting that endogenous S1P exerts a pronounced vasoconstrictor influence on afferent arteriolar tone of IR rats via S1PR2 activation. Furthermore, IR upregulated both mRNA and protein expression of S1PR2 in isolated PGMV. Importantly, the majority of sphingolipid metabolite contents including S1P were elevated in IR kidney tissue homogenates. Treatment with JTE-013 reduced plasma creatinine concentration, tubular injury and kidney ROS accumulation in IR rats. Overall, these results demonstrate that IR upregulates sphingolipid metabolites in rat kidneys and enhances renal microvascular S1P signaling via upregulation of PGMV S1PR2 expression.

One of the common features of renal hemodynamic changes after IR is increased RVR along with tubular injury and concomitant reduction of RBF and GFR, and a particularly persistent reduction of medullary blood flow (MBF).^7,50,51^ The elevated RVR could result from multiple factors such as hyperreactivity to vasoconstrictors, endothelial injury, and impairment of renal autoregulation.^32,33,52,53^ Afferent arterioles are the major renal resistance vessels controlling RBF and GFR through the adjustment of its diameter.^54^ Consistent with our previous report,^32^ the baseline diameter of afferent arterioles significantly declined in IR rats, suggesting increased RVR post-IR. We further demonstrated that IR enhanced afferent arteriolar response to exogenous S1P as evidenced by the leftward shift of the S1P vasoconstrictor profile and an 8-fold decrease in EC_50_, reflecting increased afferent arteriolar sensitivity to S1P. Nevertheless, we performed control experiments to determine whether the enhanced vasoconstriction observed in IR is S1P specific. We applied sphingosine which is the substrate for sphingosine kinases (SK) to produce S1P as the negative control agonist. Sphingosine only caused a mild afferent arteriolar vasoconstriction which was not altered by IR, implicating that S1P acts through its specific receptors.

Most biological functions of S1P are through activation of five specific G protein-coupled receptors, S1PR1-5.^15^ S1PR1, S1PR2, and S1PR3 are the predominant S1PR in cardiovascular system. Our previous study showed that S1PR1 and S1PR2 are the major S1PR expressed in PGMV.^21^ In the current study, we found that both mRNA and protein expression for S1PR2 but not for S1PR1 and S1PR3 were highly upregulated in PGMV of IR rats. Studies in mice showed that all *S1pr1, S1pr2*, and *S1pr3* mRNA levels were elevated in IR cortical tissue with a robust increase for *S1pr2*.^29^ Others however, found that mRNA expression for *S1pr1* and *S1pr3* increased in mouse IR kidneys but not for *S1pr2*.^24^ Currently, there are no data available for S1PR expression in renal microvessels of IR mice or rats. In our subsequent study, we observed a trend of increased *S1pr2* mRNA expression in cortical homogenates at 48 h post-IR while *S1pr1* and *S1pr3* mRNA remained essentially unchanged in both PGMV and cortical tissue (*n* = 5-7, Supplementary Figure 1). Collectively, our results support the upregulation of S1PR2 in PGMV of IR rats.

Several studies indicate that S1P protects against IR or cisplatin-induced kidney injury through S1PR1 activation in mice.^24-30,55^ Since activation of S1PR1 causes vasoconstriction of afferent arterioles,^21^ the enhanced S1P-mediated vasoconstriction in IR rats could reflect S1PR1 activation. The present study, however, shows that the vasoconstrictor response to SEW2871, a specific S1PR1 agonist, was similar between sham and IR rats, consistent with the unchanged mRNA and protein expression of S1PR1 in isolated PGMV. These results indicate that S1PR1 contributes little to the S1P-mediated vasoconstriction and hyperreactivity of afferent arterioles in our IR rats.

Global S1PR2 knockout mice exhibit a significantly elevated RBF compared to wild-type mice.^56^ Our previous study demonstrated that inhibition of S1PR2 activation with JTE-013 caused mild but significant vasodilation of afferent arterioles and shifted the S1P concentration-response curve to the right in rats.^21^ Those studies suggest the involvement of S1PR2 in regulating afferent arteriolar tone. In the current study, acute administration of JTE-013 dramatically increased afferent arteriolar diameter in IR rats and returned it to diameters similar to sham controls, which represents a nearly 40% increase in IR contrasted to a 9% increase in the sham group (Figure 3B). The profound impact of JTE-013 on afferent arteriolar tone in IR kidneys suggests that endogenous S1P, via S1PR2 activation, exerts a pronounced vasoconstrictor influence on afferent arteriole resistance. Combined with the mRNA and protein data on S1PR2 expression in isolated PGMV, our results indicate that the S1P-S1PR2 signaling pathway is upregulated in the PGMV of IR rats, and that the enhanced S1P-mediated vasoconstriction could contribute to the increased RVR in IR, ultimately leading to persistent reduction of RBF. This study may provide partial explanation for the studies showing the lack of beneficial effects of vasodilator therapies in combating AKI.^57,58^ and imply that RBF may have a weakened response to vasodilators. Furthermore, the enhanced S1P-induced vasoconstriction of juxtamedullary afferent arterioles could also be an important factor contributing to the vascular congestion in the renal medulla after IR^9^ caused by the medullary hypoperfusion. Therefore, a further study needs to be conducted to determine the impact of S1PR2 inhibition on renal perfusion.

Afferent arteriolar tone is regulated by a variety of vasoconstrictors.^11–13^ Following IR, many vasoconstrictors are released including ET-1 and Ang II.^59-62^ Enhanced afferent arteriolar reactivity could reflect a general increase in reactivity to any vasoconstrictors rather than being unique to S1P. ET-1 is the most potent vasoconstrictor^63^ and is elevated in plasma and kidneys after IR.^64^ Afferent arteriolar responses to ET-1, however, were unaffected in our IR rat model, consistent with the report in renal interlobular and arcuate arteries of IR rats.^65^ Besides ET-1, Ang II also plays an important role in controlling renal hemodynamics.^61^ The renin-angiotensin-aldosterone system is activated after IR.^59,62^ A large reduction of RBF in response to acute Ang II infusion was reported in rats 5-wk post-IR,^61^ suggesting enhanced sensitivity of renal vasoconstrictor responses to Ang II stimulation. Nevertheless, it remains unclear if Ang II suppresses RBF more intensely in the early stage of post-IR. Intriguingly, the afferent arteriolar response to Ang II was indistinguishable between IR and sham rats at low concentrations and may show a slight attenuation at high concentration in IR, similar to the observation in isolated afferent arterioles of IR mice.^49^ We also found normal vasoconstrictor responses to NE and KCl in IR rats,^32^ suggesting a generally intact contractile apparatus in afferent arterioles in our IR model. Overall, the current data indicate that the enhanced vasoconstriction of afferent arterioles observed in our IR rat model is not universal to all vasoconstrictors but is unique to S1P.

Growing evidence indicates that sphingolipid metabolites are elevated under a variety of pathophysiological conditions including diabetic kidneys, infarcted brain tissue, and metabolic disorders.^66-71^ Using LC/MS method, we found that the majority of sphingolipid metabolites, particularly the active sphingolipid metabolite S1P, were significantly elevated in IR kidney homogenates without changes in plasma S1P concentration. This suggests that the increased S1P content in IR kidneys is caused by local sphingolipid metabolic dysregulation, however, the mechanism that leads to elevation of select sphingolipid metabolites in post-IR kidneys remains unclear. In general, S1P is generated from sphingosine catalyzed by SK1 and/or SK2,^72,73^ and dephosphorylated to sphingosine by S1P phosphatase (SPP) but can be irreversibly degraded by S1P lyase.^74,75^ It was reported that IR selectively upregulated SK1 but not SK2 activity in mouse kidneys and inhibition of SK1 developed severe kidney injury in IR mice.^28^ In contrast, a recent study in mice indicated that IR increased local S1P released and secreted from kidney perivascular cells via SK2 activation which prompted kidney inflammation and fibrosis after IR.^76^ It is currently unclear if IR alters SK or SPP activities in rat kidneys and requires further investigation.

ROS play a critical role in S1P-mediated vasoconstriction and in IR-mediated renal microvascular dysfunction.^20,22,32^ To support the *in vitro* observation, we pretreated IR rats with JTE-013 in vivo to determine if S1PR2 blockade mitigated kidney injury. Indeed, plasma creatinine was significantly reduced in IR rats receiving JTE-013 treatment compared to IR alone. This is further confirmed by the reduction of renal ROS accumulation as demonstrated by the fluorogenic staining with H_2_DCFDA. Similar to the report in S1PR2 knockout IR mice,^29^ JTE-013-treated rats still showed necrosis and cell damage in the proximal tubules but these findings of tubule injury were significantly reduced compared to the untreated IR group. Given the severe kidney injury in this extended ischemic (60-min) rat model and the complexity of multiple factors involved in IR kidney injury, it was not surprising that two intraperitoneal injections of JTE-013 produced only partial kidney protection. The timing of the injections—30 min prior to ischemia and at the time of reperfusion—further suggest a longer intervention with JTE-013 might provide more effective renoprotection against IR. Overall, these studies suggest that S1PR2 blockade provides renoprotection against IR, consistent with S1P being involved in IR-AKI via S1PR2 activation.^29,56^

It was reported that the reductions in medullary blood flow preceded changes in overall RBF following IR and is persistent after complete recovery of the total RBF and cortical RBF.^7,77,78^ Here, we used the blood-perfused JMN preparation which is ideally suited to this study because the afferent arterioles of juxtamedullary nephrons exert a major influence in controlling medullary perfusion. While we provided compelling evidence showing that enhanced S1P-mediated vasoconstriction of juxtamedullary afferent arterioles after IR injury, it is worth mentioning that juxtamedullary nephrons represent just 10-15% of the total nephron population.^79^ Future studies will therefore be required to determine the impact of S1P on whole or regional RBF (cortical vs. medullary) in IR-AKI.

In conclusion, the current studies reveal that renal IR leads to enhanced sensitivity of juxtamedullary afferent arterioles to S1P-mediated vasoconstriction and upregulated S1PR2 expression in PGMV. IR evokes upregulation of sphingolipid metabolism in kidneys. IR-induced reduction of afferent arteriolar diameter was reversed by acute blockade of S1PR2 activation. Inhibition of S1PR2 activation improved kidney function and reduced kidney ROS accumulation and tubular injury. The hyperreactivity of afferent arterioles to S1P-mediated vasoconstriction after IR could represent a common pathophysiological mechanism leading to profound and persistent increases of RVR in IR-AKI.

## Supplementary Material

zqaf024_Supplemental_File

## Data Availability

The original data from the current studies are presented in the manuscript and the Supplementary materials and will be shared with external researchers upon requests through collaborative agreements with the corresponding author, as required by NIH data sharing policy. We will ensure that the technology (materials and methodology) remains widely available to the research community in accordance with the NIH Principles and Guidelines.
