# Supplementary material for: Sphingosine-1-Phosphate Receptor 2 Promotes Renal Microvascular Constriction and Kidney Injury Following Renal Ischemia-Reperfusion in Rats
Source: Function (Oxf). 2025 Jun 6;6(3):zqaf024. doi: 10.1093/function/zqaf024 (PMC12198761; doi:10.1093/function/zqaf024)
Supplement: zqaf024_Supplemental_File [file zqaf024_supplemental_file.docx]

**Supplemental Material**

**Sphingosine-1-Phosphate Receptor 2 Promotes Renal Microvascular Constriction and Kidney Injury Following Renal Ischemia-Reperfusion in Rats**

Zhengrong Guan^1^*, Colton E. Remedies^1^, Yanfeng Zhang^2^, Paul W. Sanders^1,3^, Edward W. Inscho^1^ and Wenguang Feng^1^

^1^ Division of Nephrology, Department of Medicine and ^2^ Department of Genetics, Heersink School of Medicine, University of Alabama at Birmingham, Birmingham, Alabama 35294

^3^ Department of Veterans Affairs Medical Center, Birmingham, Alabama, USA 35294

**Supplementary Table 1. Primers used in real-time polymerase chain reaction analyses for S1P receptors**

| **Gene** | **Forward primer** | **Reverse primer** |
| --- | --- | --- |
| *S1pr* | GCAGTTCCGAGAAGTCTCTG | CAGGATGTCACAGGTCTTCG |
| *S1pr* | CAGGATGTCACAGGTCTTCG | AGAGACAGGGAAGAAGGCTT |
| *S1pr* | CAAAGCTTCTCGTCTCCCTC | GAAGAGGGTGCAAAGAACCA |
| *18s* | ATTTGACTCAACACGGGAAA | TCGCTCCACCAACTAAGAAC |

**Supplementary Table 2. Sphingolipid metabolite contents in kidney cortical and medullary tissue homogenates and plasma of male rats 24 hours post-ischemia reperfusion (IR)**

|  | **Cortical tissue**  **(pmole/1 mg protein)** | | **Medullary tissue**  **(pmole/1 mg protein)** | | **Plasma (pmole/100 μL)** | |
| --- | --- | --- | --- | --- | --- | --- |
| **Groups** | **Sham (n = 4)** | **IR (n = 4)** | **Sham (n = 4)** | **IR (n = 4)** | **Sham (n = 4)** | **IR (n = 4)** |
| **C14-Cer** | 2.2 ± 0.5 | 4.8 ± 0.7 * | 3.2 ± 0.3 | 9.8 ± 4.1 | 1.0 ± 0.1 | 0.8 ± 0.1 |
| **C16-Cer** | 35.5 ± 3.8 | 104.3 ± 7.2 * | 48.3 ± 3.8 | 174.0 ± 4.8* | 8.0 ± 0.9 | 9.8 ± 1.3 |
| **C18-Cer** | 10.8 ± 1.7 | 22.8 ± 5.1 | 12.5 ± 1.1 | 16.9 ± 2.3 | 3.3 ± 1.2 | 9.4 ± 2.6 |
| **C18:1-Cer** | 7.2 ± 1.0 | 19.4 ± 4.8 * | 8.7 ± 1.1 | 10.3 ± 1.4 | 0.6 ± 0.2 | 1.3 ± 0.1* |
| **C20-Cer** | 36.3 ± 6.2 | 56.7 ± 5.7 | 60.0 ± 9.0 | 48.8 ± 7.9 | 6.7 ± 0.8 | 8.6 ± 1.8 |
| **C20:1-Cer** | 3.7 ± 0.8 | 5.0 ± 0.7 | 2.8 ± 0.5 | 2.4 ± 0.2 | 2.0 ± 0.3 | 1.7 ± 0.3 |
| **C20:4-Cer** | 0.10 ± 0.03 | 0.53 ± 0.15 * | 0.14 ± 0.06 | 0.22 ± 0.01 | 0.09 ± 0.02 | 0.08 ± 0.02 |
| **C22-Cer** | 67.2 ± 4.6 | 137.1 ± 18.2 * | 82.6 ± 6.2 | 147.5 ± 14.3 * | 47.4 ± 4.0 | 37.2 ± 5.5 |
| **C22:1-Cer** | 25.9 ± 1.8 | 38.1 ± 2.5 * | 16.1 ± 1.6 | 23.3 ± 3.6 | 5.0 ± 0.6 | 4.4 ± 0.6 |
| **C24-Cer** | 686 ± 24 | 797 ± 46 | 586 ± 30 | 703 ± 85 | 333 ± 12 | 262 ± 37 |
| **C24:1-Cer** | 361 ± 15 | 517 ± 29 * | 271 ± 11 | 483 ± 105 | 114 ± 7 | 82 ± 10 * |
| **C26-Cer** | 38.4 ± 3.9 | 47.7 ± 7.0 | 60.9 ± 10.7 | 67.3 ± 11.0 | 16.6 ± 2.4 | 12.6 ± 2.4 |
| **C26:1-Cer** | 10.0 ± 0.3 | 11.4 ± 1.0 | 11.2 ± 1.2 | 16.8 ± 3.8 | 8.0 ± 1.0 | 6.5 ± 1.3 |
| **dhC16-Cer** | 1.1 ± 0.2 | 4.2 ± 0.5 * | 1.6 ± 0.1 | 8.0 ± 2.1 * | 0.6 ± 0.1 | 1.0 ± 0.2 |
| **dhSph** | 1.8 ± 0.2 | 4.5 ± 0.8 * | 2.0 ± 0.3 | 6.8 ± 0.2 * | 0.3 ± 0.1 | 0.6 ± 0.2 |
| **dhS1P** | 0.29 ± 0.03 | 0.83 ± 0.14 * | 0.4 ± 0.1 | 1.2 ± 0.1 * | 15.8 ± 2.0 | 18.3 ± 2.5 |
| **Sph** | 19.5 ± 0.7 | 36.0 ± 3.5* | 22.5 ± 3.3 | 40.8 ± 2.5 * | 0.9 ± 0.1 | 1.9 ± 0.6 |
| **S1P** | 1.2 ± 0.1 | 3.2 ± 0.5 * | 1.8 ± 0.4 | 3.7 ± 0.5 * | 91 ± 10 | 77 ± 8 |

Supplementary Table 2 shows a total of 18 sphingolipid metabolites measured in renal cortical and the outer medullary tissue homogenates and plasma collected from male sham-operation rats (sham) and rats subjected ischemia followed by 24 hours of reperfusion (IR). Values are expressed as means ±SEM. Comparisons between groups were performed with unpaired t test. **P* < 0.05 vs. sham for each metabolite. n = 4/each group. Cer: ceramide; dhSph: dihydrosphingosine; dhS1P: dihydrosphingosine-1-phosphate; Sph: sphingosine; S1P: sphingosine-1-phosphate.


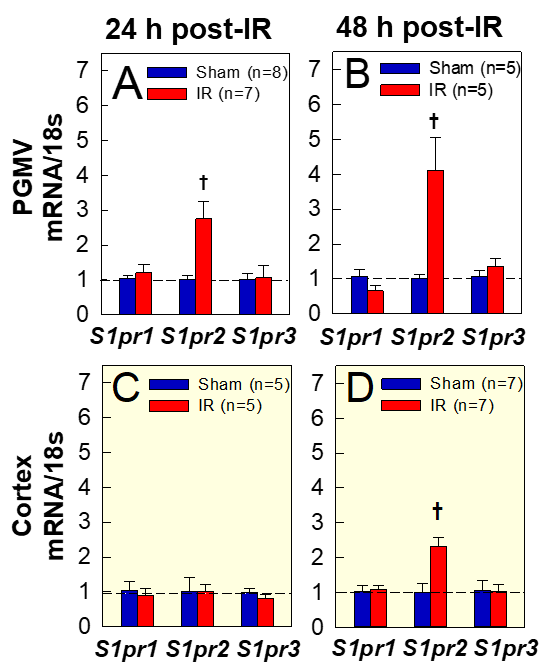
**Supplementary Figure 1**

**sFigure 1. mRNA expression of S1P receptors (*S1pr*) 1, 2 and 3 in isolated preglomerular microvessels (PGMV) and renal cortical tissue homogenates of sham and rats subjected ischemia followed by 24 or 48 hours of reperfusion (IR).**

The total mRNA of *S1pr2* was increased significantly in PGMV isolated from rats at 24 hours post-IR (A) and continuously elevated at 48 hours (B) whereas the mRNA levels of *S1pr1* and *S1pr3* remained unchanged. The mRNA levels of all three *S1pr* were unaltered in cortical homogenates of IR rats at 24 hours (C) but *S1pr2* mRNA level was elevated at 48 hours post-IR (D). Values are means ± SE. Comparison between groups was performed with unpaired t test. †*P* < 0.05 vs. sham for each S1PR. *n* represents the numbers of rats.
